# Sunitinib or Pazopanib: Is There Any Difference Between Tyrosine Kinase Inhibitors in the Pre-Nivolumab Setting in Metastatic Renal Cell Carcinoma?

**DOI:** 10.7759/cureus.10525

**Published:** 2020-09-18

**Authors:** Gokhan Ucar, Yusuf Acikgoz, Yakup Ergun, Oznur Bal, Mesut Yilmaz, Serdar Karakaya, Nadiye Akdeniz, Osman Kostek, Ozlem Aydin Isak, Gorkem Yazici Sener, Merve Dirikoc, Selin Aktürk Esen, Mutlu Dogan, Dogan Uncu

**Affiliations:** 1 Medical Oncology, Health of Science Ankara City Hospital, Ankara, TUR; 2 Medical Oncology, Bakırkoy Sadi Konuk Training and Research Hospital, Istanbul, TUR; 3 Medical Oncology, Health of Science Abdurrahman Yurtaslan Training and Research Hospital, Ankara, TUR; 4 Medical Oncology, Dicle University Medical Faculty, Diyarbakır, TUR; 5 Medical Oncology, Trakya University Medical Faculty, Edirne, TUR; 6 Medical Oncology, Health of Science Diskapi Yildirim Beyazit Training and Research Hospital, Ankara, TUR; 7 Internal Medicine, Gazi University Medical Faculty, Ankara, TUR

**Keywords:** renal cell carcinoma, metastasis, sunitinib, pazopanib, nivolumab

## Abstract

Introduction

Treatment options for metastatic renal cell carcinoma disease have been improved in recent years. However, there is still no optimal treatment sequence or combination for metastatic disease. We aimed to investigate whether patients differed in terms of disease outcomes regarding pre-nivolumab tyrosine kinase inhibitors (TKIs).

Material and methods

The analysis of patients was performed after all cohorts were sub-grouped into two groups according to pre-nivolumab TKIs as following the sunitinib arm and the pazopanib arm.

Result

A total of 75 patients were included in this study. The median follow-up time was eight months for all cohorts. The objective response rate was statistically significantly higher in the pazopanib arm as compared to the sunitinib arm (56% vs 30%, p=0.02). Progression-free survival was significantly higher in pazopanib than sunitinib (10.3 months vs 5.3 months, p=0.02). Multivariate analysis revealed that pazopanib treatment was associated with better progression-free survival (HR: 0.44, 95 CI; 0.22-0.91, p=0.02). While the median overall survival for patients who had received sunitinib was 11.0 months, it has not been reached the median in the pazopanib arm (11.0 months vs NR, p=0.051).

Discussion

We demonstrated significantly better progression-free survival and a higher objective response rate with nivolumab treatment in patients who had received pazopanib as compared with patients who received sunitinib in the pre-nivolumab period.

## Introduction

Kidney and renal pelvis carcinoma are estimated to constitute approximately 4.2% of all new cancer cases and 2.4% of all cancer-related deaths in 2019 [1]. Of those, approximately 16% of patients are diagnosed at stage IV and the estimated five-year survival rate is only 12% [1]. The majority of kidney cancers are composed of renal cell carcinoma (RCC), with a ratio of 85%. Clear cell histology comprises 70% of renal cell carcinoma cases [2-4].

Surgery is still the mainstay of the treatment for patients with localized disease [5]. However, treatment options for metastatic RCC (mRCC) have shifted from interleukin to newer targeted therapies and immunotherapies in the last decade. Before the era of targeted therapies, the median overall survival of patients with mRCC was approximately one year [6-7]. The vascular endothelial growth factor receptor tyrosine kinase inhibitors (VEGFR-TKIs) have been one of the standards of care for the management of patients with mRCC. Of those, sunitinib and pazopanib are the preferred TKIs for first-line treatment. Recently, immune checkpoint inhibitors (ICI) have been introduced into the management of mRCC, with a substantial achievement either alone or in combination with TKIs.

Although sunitinib and pazopanib have demonstrated similar efficacy in the first-line settings, they had differences in terms of side effects and tolerability. Furthermore, it is still not known whether they have any impact on the outcomes of following ICI treatments. In the literature, there are various studies giving indirect information about the influence of TKIs on the following treatments. However, there is still no suggestion about the selection of any of the TKIs in first-line settings.

In this retrospective multicenter study, we aimed to investigate whether patients who had received sunitinib or pazopanib differed in terms of disease outcomes with nivolumab treatment in mRCC.

## Materials and methods

Patient characteristics

We retrospectively evaluated the data of 286 patients who were admitted to seven different oncology centers. Of those, 75 patients who received nivolumab due to progression on treatment with either sunitinib or pazopanib were included in this study. The analysis of patients was performed after all cohorts were sub-classified into two groups according to pre-nivolumab TKIs as following the sunitinib arm and the pazopanib arm.

The inclusion criteria were as follows: i) aged at or over 18 years, ii) histopathologically proven clear cell renal cell carcinoma, iii) Eastern Cooperative Oncology Group (ECOG) performance score (PS) ≤2, iv) measurable metastatic disease according to Immune Response Evaluation Criteria in Solid Tumors (iRECIST) criteria, v) adequate liver, kidney, and bone marrow function. Patients with brain metastasis, non-clear cell histology, and those who had received Interferon-alfa for more than three months were excluded from the study.

The following data were obtained from patients' oncologic follow-up files: age, gender, ECOG performance score, International Metastatic RCC Database Consortium (IMDC) risk score, presence of cytoreductive nephrectomy, duration of metastatic disease, number of metastatic sites, previous treatment and duration, and the results of the biochemical analysis performed before nivolumab treatment.

All patients had received Interferon-alfa (IFN-ɑ) after the diagnosis of metastatic settings due to the restriction of health policies in our country. All patients received either sunitinib (50 mg, 4 weeks on - 2 weeks off) or pazopanib (800 mg/day, continuously) after IFN-ɑ treatment due to unacceptable toxicity. Patients who progressed on sunitinib or pazopanib were treated with nivolumab 3 mg/kg every two weeks. All patients underwent physical examination, hematological, biochemical, and hormonal (thyroid-stimulating hormone (TSH), cortisol) evaluation every two weeks. Tumor response was evaluated by computed tomography every 12 weeks according to iRECIST criteria.

According to immune RECIST criteria, complete response (CR) included the disappearance of all target lesions and reduction in the short axis measurement of all pathologic lymph nodes to ≤10 mm, partial response (PR) was defined as a ≥30% decrease in tumor size, progressive disease (PD) was defined as a ≥20% increase in tumor size, and stable disease (SD) was considered for patients who met neither PR nor PD criteria. The objective response rate (ORR) was defined as the sum of complete and partial responses. The disease control rate (DCR) was defined as the sum of the complete response, partial response, and stable disease rates. Hyperprogression was defined as a two-fold increase of tumor burden at first evaluation for nivolumab treatment and the presence of symptoms related to progression. The detection of new lesions was not considered as progression, and comparisons were performed after new lesions were added to the total tumor burden measured before treatment [8].

Univariate and multivariate analysis

For univariate and multivariate analysis, seven variables that could have an impact on progression-free survival were selected based on previous studies. Variables were categorized into two groups as follows: age (<65 years or ≥65 years), gender (male or female), ECOG PS (0-1 or 2), number of metastatic sites (single or multiple), bone metastasis (present or absent), liver metastasis (present or absent), and lung metastasis (present or absent). Variables with a p-value of <0.05 were included in the multivariate analysis.

Statistical analysis

Statistical analysis was performed by using Statistical Package for the Social Sciences Version 22.0 for Windows (IBM Corp, Armonk, NY). Patients’ characteristics and response rates were compared by the Pearson chi-square test and Fisher’s exact test. The Kaplan-Meier test was used for survival analysis, and the survival outcomes were analyzed by the log-rank test. The Cox proportional-hazards model was applied for the multivariate analysis. All reported p-values were two-sided and p-values of less or equal than 0.05 were considered to as a significant result.

The overall survival was defined as the time between the date of starting nivolumab to death or the last visit for living patients. Progression-free survival was defined as the time between the date of starting nivolumab treatment to disease progression or death, whichever occurred first.

## Results

A total of 75 patients were included in this study. Of those, 41 patients were in the sunitinib arm and 34 patients were in the pazopanib arm. The median age was 59 years (range: 26-78) for all patients, and it was 59 years in both arms. The baseline characteristics of both groups were similar to each other except for the rate of bone and lung metastases. The ratio of patients with bone metastasis was higher in the pazopanib arm than in patients in the sunitinib arm (53% vs 27%, p=0.02, respectively). The baseline characteristics of both groups were summarized in Table 1.

**Table 1:** Baseline characteristics ECOG: Eastern Cooperative Oncology Group, IMDC: International Metastatic RCC Database Consortium

	Sunitinib (n:41)	Pazopanib (n:34)	p-value
Age, median (range)	59 (26-78)	59 (28-74)	0.912
<65years	21(51%)	17(50%)	
≥65years	20(49%)	17(50%)	
Sex			0.825
Male	28 (68%)	22 (65%)	
Female	13 (32%)	12 (35%)	
IMDC risk score at diagnosis			0.835
Favorable	10 (24%)	9 (26%)	
Intermediate-Poor	31 (76%)	25 (74%)	
Cytoreductive nephrectomy at diagnosis			0.081
No	28 (68%)	29 (85%)	
Yes	13 (32%)	5 (55%)	
ECOG performance status			0.763
0-1	33 (80%)	26 (77%)	
.2	8 (20%)	8 (23%)	
Number of metastatic sites			0.217
Single	19 (46%)	11 (32%)	
Multiple	22 (54%)	23 (68%)	
Bone metastasis			0.026
Yes	11 (27%)	18 (53%)	
No	30 (73%)	16 (47%)	
Liver metastasis			0.636
Yes	8 (20%)	5 (15%)	
No	33 (80%)	29 (85%)	
Lung metastasis			0.045
Yes	25 (61%)	28 (82%)	
No	16 (39%)	6 (18%)	
Neutrophil lymphocyte ratio (median)	2.9	3.2	0.549

The median duration from the diagnosis of metastatic disease to starting nivolumab treatment was 21.1 months for patients in the sunitinib arm, whereas it was 18.9 months for patients in the pazopanib arm. The median duration of interferon-alfa treatment was 1.6 months and 1.0 months for the sunitinib and pazopanib arms, respectively. While the median duration of TKI treatment was 8.7 months in the sunitinib arm, it was 9.8 months for pazopanib treatment. Objective response rates were 30% and 46% for sunitinib and pazopanib treatments in the pre-nivolumab period, respectively. Table 2 demonstrates the treatment results that were achieved with sunitinib and pazopanib treatments given in the pre-nivolumab period.

**Table 2:** Characteristics of pre-nivolumab treatments

	Sunitinib	Pazopanib
Median duration of metastatic disease (months)	21.1	18.9
Median duration of first-line treatment (months)	2.6	2.0
Response rates of first-line treatment		
Complete response rate	5%	─
Partial response rate	22%	30%
Objective response rate	27%	30%
Stable disease rate	25%	15%
Progressive disease rate	48%	55%
Median duration of second-line treatment (months)	8.7	9.8
Response rates of second-line treatment		
Complete response rate	─	3%
Partial response rate	30%	43%
Objective response rate	30%	46%
Stable disease rate	30%	20%
Progressive disease rate	40%	34%

The median follow-up time was 8.0 months for all cohorts. Complete response was observed in two patients (3%) and partial response in 30 patients (40%) in all patients. Complete response was observed in one patient with nivolumab treatment in both the sunitinib and pazopanib arms. However, the rate of partial response was statistically significantly higher in the pazopanib arm as compared to the sunitinib arm (53% vs 28%, respectively). Response rates are summarized in Table 3.

**Table 3:** Tumor response rates for nivolumab treatment

	Sunitinib	Pazopanib	p-value
	(n: 41)	(n: 34)	
Complete response	1 (2%)	1 (3%)	0.925
Partial response	12 (28%)	18 (53%)	0.023
Objective response rate	13 (30%)	19 (56%)	0.028
Stable disease	11 (25%)	10 (29%)	0.715
Disease control rate	24 (55%)	29 (85%)	0.005
Progressive disease	20 (45%)	5 (15%)	0.005

**Figure 1:**
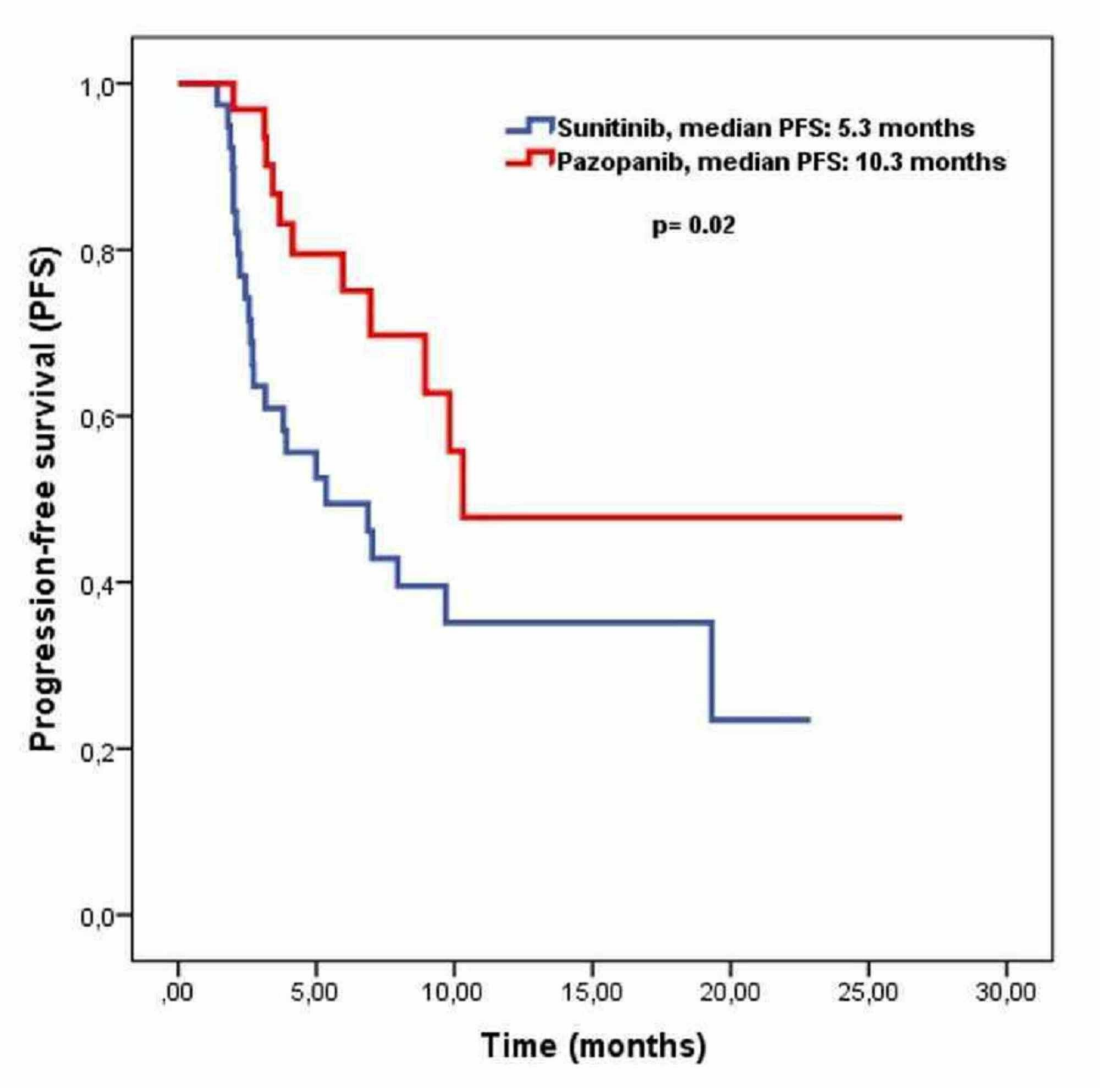
Progression-free survival curves for patients used sunitinib or pazopanib

Median progression-free survival was 8.9 months (95% CI; 5.9-11.9) for all cohorts. Progression-free survival was significantly higher in the pazopanib arm than the sunitinib arm (10.3 months vs 5.3 months, p=0.02) (Figure 1).

Univariate analysis, which was performed through determining progression as the endpoint, showed that ECOG PS 0-1 and absence of liver metastasis were associated with longer progression-free survival. Further analysis of variables that had a statistically significant association with progression-free survival in the univariate analysis revealed that pazopanib as pre-nivolumab treatment was an independent prognostic factor for progression-free survival (HR: 0.44, 95 CI; 0.22-0.91, p=0.02) (Table 4).

**Table 4:** Univariate and multivariate analysis results for progression-free survival PFS: Progression-free survival, ECOG: Eastern Cooperative Oncology Group

Variable	Univariate analysis			Multivariate analysis	
	Median PFS (months)	p-value		HR, (95 % CI)	p-value
Age (years)	9.6 vs. 8.9	0.815			
<60 vs. ≥60					
Sex	9.8 vs. 8.9	0.526			
Female vs. Male					
ECOG performance status	15.3 vs. 5.9	0.007		0.41, (0.20-0.82)	0.016
0-1 vs. 2					
Number of metastatic sites	10.3 vs. 7.9	0.312			
Single vs. multiple					
Bone metastasis	8.9 vs. 9.8	0.824			
Yes vs. No					
Liver metastasis	3.4 vs. 10.3	0.021		0.52, (0.24-1.14)	0.125
Yes vs. No					
Lung metastasis	8.0 vs. 9.9	0.412			
Yes vs. No					
Pre-nivolumab	5.3 vs. 10.3	0.021		0.44, (0.22-0.91)	0.029
Sunitinib vs. pazopanib					

While the median overall survival for patients received sunitinib as pre-nivolumab TKI was 11.0 months, it has not reached the median in the pazopanib arm (11.0 months vs NR, p=0.051). Survival analysis could not be performed because survival data were immature at the time of the final analysis.

Hyperprogession was observed in three patients and all of those cases were in the sunitinib arm. Nivolumab was permanently discontinued in two patients due to prolonged grade 4 hematologic toxicity (neutropenia) and grade 4 neurological toxicity (encephalitis) in the pazopanib arm. In the sunitinib arm, nivolumab had to be discontinued in one patient due to grade 4 nephropathy (proteinuria).

## Discussion

There are various studies demonstrating the effectiveness of sunitinib and pazopanib in first-line settings and nivolumab in second-line settings for mRCC. However, to the best of our knowledge, there is no study directly comparing the impact of TKIs on subsequent immunotherapy. This is the first study performing this evaluation in a head-to-head fashion. In this study, we evaluated the influence of pre-nivolumab TKIs on the following nivolumab treatments. After a median eight-months follow-up, our findings showed that patients who had received pazopanib before nivolumab achieved better progression-free survival as compared to patients who had received sunitinib in the pre-nivolumab period (10.3 months vs 5.3 months, p=0.02). There was no statistically significant difference in terms of baseline characteristics, except for bone and liver metastasis, between the sunitinib arm and the pazopanib arm. Additionally, patients who had received pazopanib as pre-nivolumab TKI achieved higher partial response rates compared to patients who had received sunitinib (53% vs 28%, respectively). On the other hand, we could not perform overall survival analysis due to it not reaching the median. Taken together, patients who received pazopanib as pre-nivolumab TKI experienced better progression-free survival with nivolumab treatment as compared to patients who had received sunitinib before nivolumab treatment.

The first trial demonstrating a head-to-head comparison of sunitinib and pazopanib in mRCC as first-line treatment was the COMPARZ (Pazopanib Versus Sunitinib in the Treatment of Locally Advanced and/or Metastatic Renal Cell Carcinoma) study. The primary endpoint of this phase 3, prospective randomized study was to show the non-inferiority of pazopanib compared to sunitinib regarding progression-free survival. According to the result of this study, median progression-free survival was 9.5 months for sunitinib and 8.4 months for pazopanib. Although progression-free survival for the sunitinib arm was numerically longer, the hazard ratio for progression or death was 1.05 (95% CI: 0.90-1.22), which was lower than the predefined upper bound margin for non-inferiority [9]. In 2014, the overall survival data of this trial demonstrated no significant difference in terms of OS between sunitinib and pazopanib (29.1 months vs 28.3 months, respectively)[10]. Furthermore, a cross-over study (PISCES - Assessing Treatment Preference for Pazopanib Versus Sunitinib in Patients With Metastatic Renal Cell Carcinoma - trial) showed a higher patient preference for pazopanib over sunitinib (70% vs 22%) due to a better overall quality of life [11]. These data give us a prediction about the impact of the quality of life on the selection of drugs with similar activity.

The phase 3 Checkmate-025 (Study of Nivolumab (BMS-936558) vs. Everolimus in Pre-Treated Advanced or Metastatic Clear-cell Renal Cell Carcinoma) trial has obviously demonstrated the superiority of nivolumab over everolimus in patients with mRCC who progressed on one or two anti-angiogenic TKI. This study showed that overall survival for patients who received nivolumab was 25.0 months and for patients who received everolimus was 19.6 (p<0.001) [12]. Additionally, the objective response rate was significantly higher in nivolumab as compared with everolimus (25% vs 5%, p<0.001) [12]. Recently, the Checkmate-016 trial aimed to evaluate the efficacy of nivolumab in combination with either sunitinib or pazopanib in patients with mRCC. According to the result of this study, patients in nivolumab plus sunitinib achieved higher progression-free survival as compared with patients in nivolumab plus pazopanib (12.7 months vs 7.2 months, respectively). However, nivolumab plus the sunitinib arm consisted of a relatively higher rate of treatment-naive patients than nivolumab plus the pazopanib arm. Additionally, adverse event rates were significantly higher in both groups. A higher rate of toxicity and an unbalanced cohort of two groups did not allow to translate these combined treatment modalities into clinical practice [13]. This study suggests a sequential treatment modality rather than a combined treatment of TKIs and nivolumab.

The impact of prior anti-angiogenic treatment on subsequent treatments has been evaluated in previous studies indirectly. After patients were stratified according to prior treatments, the analysis of the Checkmate-025 trial showed that while median overall survival for patients who received sunitinib as pre-nivolumab TKI was 23.6 months, it has not reached the median for patients who received pazopanib as prior nivolumab treatment. Although there was no direct comparison between those two groups, the authors did not report any difference in overall survival regarding prior TKIs. Notably, the SUNPAZ (A Phase 4 Study of Everolimus to Evaluate Efficacy and Safety in Patients with Metastatic Renal-Cell Carcinoma after Failure of First-Line Sunitinib or Pazopanib) study had an appropriate study design for evaluating the influence of sunitinib and pazopanib on subsequent treatment [14]. The SUNPAZ trial is a phase 4, multicenter, prospective study that aimed to evaluate the efficacy and safety of everolimus in patients who progressed on prior anti-angiogenic treatments (sunitinib or pazopanib) in mRCC [14]. According to the result of this study, the median progression-free survival for patients in the sunitinib group was 2.8 months, whereas it was eight months in the pazopanib group. Additionally, overall survival was shorter in sunitinib than in pazopanib (14.7 months vs 20.4 months). Although this study claimed the positive impact of pazopanib on the following treatments, as authors stated a lower number of patients (n:25) for all cohorts should be taken into consideration. The METEOR (A Study of Cabozantinib (XL184) vs Everolimus in Subjects With Metastatic Renal Cell Carcinoma) trial aimed to evaluate the efficacy of cabozantinib in patients who progressed at least one line TKIs in mRCC. Further analysis of clinical outcomes by prior anti-angiogenic treatments showed that median overall survival for patients who received sunitinib as prior treatment was 21.4 months with cabozantinib, whereas it was 22 months in those who received pazopanib as prior anti-angiogenic TKI [15]. Similar to previous studies, there was no direct comparison between sunitinib and pazopanib.

Although we do not know the exact mechanism of difference observed in those two groups, according to an in vitro study, sunitinib has been shown to reduce myeloid-derived suppressor cells, which in turn modulate the immune response [16]. Additionally, Zizzari et al. conducted a cell-line study that gives important information about the immune-regulatory effects of pazopanib and sunitinib [17]. In this study, human monocyte-derived dendritic cells gathered from the peripheral blood mononuclear cells of healthy donors were treated with either pazopanib or sunitinib after completion of maturation. Dendritic cells (DCs) treated with pazopanib had increased expression of CD40 and other activation markers than those treated with sunitinib. Furthermore, pazopanib treatment caused a decreased level of PD-L1 expression as compared with sunitinib treatment. Consequently, pazopanib has been shown to increase the immune response more efficiently through activating DCs as compared with sunitinib. We believe that differences in response to nivolumab treatment between sunitinib and pazopanib arm may be explained by the higher activation DC potential of pazopanib than sunitinib. In addition, the failure of the pazopanib arm compared to the sunitinib arm in the Checkmate-016 trial might be interpreted as an indicator of differences in the immunomodulatory activities of those two TKIs.

The major limitations of our study are its retrospective nature, relatively low number of patients, and the absence of overall survival data.

## Conclusions

Taken together, to the best of our knowledge, our study is the first study reporting a direct comparison of the influence of prior TKIs (sunitinib vs pazopanib) on subsequent nivolumab treatment. We demonstrated significantly better progression-free survival with nivolumab in patients who had received pazopanib in the pre-nivolumab period compared with patients who had received sunitinib as prior treatment. Our study may guide clinicians for selection between these two TKIs in countries that require utilizing one-line TKI before immunotherapy. Therefore, we believe our results need to be confirmed by further prospective clinical trials with a larger number of patients.
